# Bisecting N-Acetylglucosamine on EGFR Inhibits Malignant Phenotype of Breast Cancer via Down-Regulation of EGFR/Erk Signaling

**DOI:** 10.3389/fonc.2020.00929

**Published:** 2020-06-16

**Authors:** Lanming Cheng, Lin Cao, Yurong Wu, Wenjie Xie, Jiaqi Li, Feng Guan, Zengqi Tan

**Affiliations:** Shaanxi Provincial Key Laboratory of Biotechnology, Joint International Research Laboratory of Glycobiology and Medicinal Chemistry, College of Life Science, Northwest University, Xi'an, China

**Keywords:** MGAT3, bisecting GlcNAc, glycopeptide, breast cancer, EGFR, EGFR/Erk signaling

## Abstract

Glycosylation, the most prevalent and diverse post-translational modification of protein, plays crucial biological roles in many physiological and pathological events. Alteration of N-glycan has been detected during breast cancer progression. Among the specific N-glycan structures, bisecting N-Acetylglucosamine (GlcNAc) is a β1,4-linked GlcNAc attached to the core β-mannose residue, and is catalyzed by glycosyltransferase MGAT3. Bisecting GlcNAc levels were commonly dysregulated in different types of cancer. In this study, we utilized mass spectrometry and lectin microarray analysis to investigate aberrant N-glycans in breast cancer cells. Our data showed the decreased levels of bisecting GlcNAc and down-regulated expression of MGAT3 in breast cancer cells than normal epithelial cells. Using PHA-E (a plant lectin recognizing and combining bisecting GlcNAc) based enrichment coupled with nanoLC-MS/MS, we analyzed the glycoproteins bearing bisecting GlcNAc in various breast cancer cells. Among the differentially expressed glycoproteins, levels of bisecting GlcNAc on EGFR were significantly decreased in breast cancer cells, confirmed by immunostaining and immunoprecipitation. We overexpressed MGAT3 in breast cancer MDA-MB-231 cells, and overexpression of MGAT3 significantly enhanced the bisecting N-GlcNAc on EGFR and suppressed the EGFR/Erk signaling, which further resulted in the reduction of migratory ability, cell proliferation, and clonal formation. Taken together, we conclude that bisecting N-GlcNAc on EGFR inhibits malignant phenotype of breast cancer via down-regulation of EGFR/Erk signaling.

## Introduction

Breast cancer (BCa) is one of the most common cancers and associated with high mortality rates in women (1, 2). BCa detected in early stages is treatable, and the 5-year survival rate approaches 100% for the patients diagnosed at stage I, but decreases to 26% for patients diagnosed at stage IV (3). Thus, blood biomarkers of BCa for early detection is widely studied for improvement of prognosis and survival rate. Some blood-borne tumor markers were widely used for screening, monitoring, and prognosis of BCa patients, for example, carbohydrate antigen 15-3 (CA15-3) (4, 5) and carcinoembryonic antigen (CEA) (6). Notably, numerous studies focused on relevant dysregulated glycosylation in the development and progression of BCa (7, 8).

Glycosylation is a template-free enzymatic process that adds monosaccharides to biomoleculars such as carbohydrates, lipids, and proteins by glycosidic linkages to form glycoconjugates. Glycosylation play essential role in biological processes including cell-cell interaction (9), cancer metastasis (10), cell growth (11), cell adhesion (12), and host-virus interaction (13), etc. Alterations of glycosylation and glycogenes are closely associated with many disease progressions or identified as potential biomarkers for certain cancers (14, 15).

Not surprised, aberrant glycosylation has been implicated in BCa. Globo H and sialyl-Tn, which are highly expressed on BCa cells, are two typical cancer-associated carbohydrate antigens, and are good candidates for high-sensitivity diagnostics and cancer vaccine development (16–18). Recent studies have documented that elevated levels of sialylation, sLeX epitopes, and fucosylation of N-glycan were detected in serum of BCa patients (19, 20). Significant higher levels of bi-, tri-, and tetra-antennary glycans with sLeX epitopes were detected from sera of BCa patients with circulating tumor cells (CTCs) ≥5/7.5 mL compared to patients with CTCs <5/7.5 mL (21). Gangliotetraosylceramide (Gg4) and its gene β3GalT4 were downregulated in transforming growth factor-β1 (TGFβ1) induced epithelial-mesenchymal transition (EMT) of NMuMG cells (22, 23). Also, we previously observed decreased levels of bisecting N-acetylglucosamine (GlcNAc) and its glycosyltransferase N-acetylglucosaminyltransferase III (GlcNAcT- III, also termed as MGAT3) expression in TGFβ- and hypoxia- induced EMT process (24, 25).

Bisecting GlcNAc is a GlcNAc attached in a β1,4 linkage to the core β-mannose residue catalyzed by MGAT3. The presence of bisecting GlcNAc inhibits trimming by α-mannosidase II, thereby generating hybrid structures, and also prevents the actions of GlcNAcT-II, GlcNAcT-IV, and GlcNAcT-V to process and elongate N-glycan chains. Bisecting GlcNAc levels were commonly dysregulated in cancers. For examples, bisecting GlcNAc on membrane proteins were differentially expressed in metastatic, moderately differentiated, and poorly differentiated colorectal cancer cells (26). Bisecting GlcNAc structures were identified in serous and endometrioid ovarian cancer tissues, but not found in control non-malignant samples (27). Bisecting GlcNAc on E-cadherin could prolong its turnover on cell surface without affecting its expression, stabilize the E-cadherin-catenin complex, and inhibit β-catenin translocation from plasma membrane into the cytoplasm and nucleus (25). Although the bisecting GlcNAc are bound on various proteins, the systemic study on the identification of glycoproteins bearing bisecting GlcNAc and exploration of the functions of bisecting GlcNAc is not well-elucidated.

In this study, we investigated the levels of bisecting GlcNAc in various breast cells using high-throughput techniques (MALDI-TOF/TOF-MS and lectin microarray), identified the target proteins bearing bisecting GlcNAc using lectin PHA-E enrichment coupled with NanoLC-MS/MS analysis, and explored the effects of bisecting GlcNAc on target protein.

## Materials and Methods

### Cell Culture

Human mammary epithelial cell line (MCF10A) and human BCa cell lines (MCF7, MDA-MB-231, and SK-BR-3) were purchased from the Cell Bank at the Chinese Academic of Science (Shanghai, China). BCa cells were cultured in DMEM supplemented with 10% FBS (Biological Industries; Kibbutz Beit Haemek, Israel), 100 UI/mL penicillin, and 100 μg/mL streptomycin (Gibco; Carlsbad, CA, USA). MCF10A cells were grown in DMEM/F12 supplemented with 100 ng/mL cholera enterotoxin, 10 μg/mL insulin, 0.5 μg/mL hydrocortisol, 20 ng/mL EGF, 5% horse serum, 100 UI/mL penicillin, and 100 μg/mL streptomycin at 37°C in 5% CO_2_.

### Total Protein Extraction

Total protein extraction was performed as described previously (24). Briefly, cells were detached, rinsed, and lysed with RIPA buffer (50 mM Tris, pH 7.2, 1% Triton X-100, 0.5% sodium deoxycholate, 0.1% SDS, 150 mM NaCl, 10 mM MgCl_2_, 5% glycerol) supplemented with protease inhibitor, and centrifuged. The supernatant was collected and stored at −80°C. Protein content was determined by BCA assay (Beyotime Institute of Biotechnology; Haimen, China).

### Western Blot

Western blot was performed as described previously. In brief, the total protein (30 μg) were separated SDS-PAGE. The gels were transferred to PVDF membranes. The membranes was blocked with 5% BSA, and incubated with primary antibodies against MGAT3 (1:500; ab135514; Abcam, Cambridge, MA, UK), GAPDH (1:10,000; G9545; Sigma-Aldrich, St. Louis, MO, USA), ERK (1:1,000; 4696; Cell Signaling Technology, Beverly, MA, USA), p-ERK (1:1,000; 4370; Cell Signaling Technology), AKT (1:1,000; 4685; Cell Signaling Technology), p-AKT (Ser473; 1:2,000; 4060; Cell Signaling Technology), p-EGFR (Y1173; 1:1,000; 4407; Cell Signaling Technology), p-EGFR (Y1086; 1:1,000; 3777; Cell Signaling Technology) and EGFR (1:1,000; 4267; Cell Signaling Technology) overnight at 4°C, and incubated with HRP conjugated secondary antibody.

### MALDI-TOF/TOF-MS of N-glycans From Breast Cell Lines

MALDI-TOF/TOF-MS of N-glycans were performed as described previously (24). Briefly, cell lysates were denatured with UREA, DTT and IAM, and digested with PNGase F (New England BioLabs; Ipswich, MA, USA). Released N-glycans were collected, desalted with HyperSep Hypercarb SPE cartridges (Thermo Fisher Scientific, Bellefonte, PA, USA), lyophilized, and subjected to MALDI-TOF/TOF-MS (UltrafleXtreme, Bruker Daltonics; Bremen, Germany) analysis in positive-ion mode. Mass signals were analyzed and annotated with GlycoWorkbench software (http://code.google.com/p/glycoworkbench) (28). Relative proportion was calculated by adding the relative intensity of the same type of N-glycans.

### Lectin Microarray Analysis

Lectin microarray was performed and analyzed as described previously (29). In brief, 37 lectins from Vector Laboratories (Burlingame, CA, USA), Sigma-Aldrich (St. Louis, MO, USA), or Merck (Darmstadt, Germany) were immobilized onto epoxysilane-coated solid support. Sample proteins were labeled with Cy3 (GE Healthcare; Buckinghamshire, UK) and subjected to the lectin microarray. The microarrays were scanned with a GenePix 4000B confocal scanner (Axon Instruments; Union City, CA, USA). The data was analyzed as previously described (29).

### Lectin Staining/Immunofluorescence

Lectin staining was performed as described previously (29). In brief, cells cultured in confocal dishes, were rinsed with ice-cold 1 × PBS, fixed with 4% paraformaldehyde, permeabilized with 0.2% Triton X-100, blocked with 5% BSA. For lectin staining, cells were incubated with FITC-labeled PHA-E (FL-1121; Vector Laboratories, Burlingame, CA, USA) and SNA (FL-1301; Vector Laboratories). For immunofluorescence, cells were incubated with primary antibodies against EGFR (D38B1; Cell Signaling Technology; Danvers, MA, USA) and fluorescence-labeled secondary antibody. Cells were stained with DAPI, rinsed with PBS, and photographed with confocal microscope (Olympus FV1000; Olympus; Tokyo, Japan).

### Protein Digestion

Protein digestion was performed as described previously (30). In brief, proteins were extracted with 8 M UREA/1 M NH_4_HCO_3_ from above cells and sonicated thoroughly. Total proteins (2 mg) were reduced by 5 mM DTT for 1 h at 37°C, alkylated by addition of 15 mM IAM for 30 min in the dark at RT, and incubated with sequencing grade trypsin (Promega; Madison, WI, USA; protein: enzyme, 50:1, w/w) at 37°C overnight. The digested peptides were acidified with 10% trifluoroacetic acid to pH < 3 and desalted with Oasis HLB SPE column (Waters; Milford, MA, USA).

### Glycoprotein Enrichment by Lectin PHA-E

Total peptides (150 μg) were incubated with agarose bound PHA-E in binding buffer (20 mM Tris-HCl containing 100 mM NaCl, 5 mM MnCl_2_, and 5 mM CaCl_2_) overnight at 4°C. The mixture was centrifuged for 1 min at 2,500 g and supernatant was removed. The precipitates were rinsed four times with binding buffer by vortex for 10 s. The sample was boiled for 10 min, and supernatant was collected after centrifugation. Glycopeptides were digested with PNGase F and lyophilized.

### NanoLC-MS/MS Analysis

The enriched glycopeptides were analyzed by an Orbitrap Fusion Lumos mass spectrometer (Thermo Fisher Scientific). Peptides were separated on an Easy-nLC 1200 system with a 20 cm C18 separating column. Mobile phase flow rate was 300 nL/min and consisted of 0.5% formic acid in water (A) and 0.1% formic acid in acetonitrile (B). The gradient profile was set as follows: 0–12% B for 10 min, 12–33% B for 130 min, 33–100% B for 15 min, and 100% B for 10 min. The spray voltage was set at 2,000 V. Orbitrap spectra (AGC 1 × 10^6^) was collected from 300 to 2,000 m/z at a resolution of 60 K using an isolation window of 0.7 m/z. A dynamic exclusion time of 10 s was used. All LC-MS/MS data was searched against reviewed Homo sapiens reference proteome databases (version 201502) using Maxquant 1.5.1.12 (Cox, Mann, 2008). The sequences and masses of peptides were extracted from the results with FDR <1%.

### Immunoprecipitation

Cell lysates (500 μg) were incubated with 1 μg primary antibody against EGFR (D38B1) for 2 h at 4°C, and 20 μL protein A/G-agarose (Santa Cruz Biotechnology; Santa Cruz, CA, USA) was added. The mixture was incubated with rotation overnight at 4°C, rinsed with PBS, denatured with loading buffer, and subjected to SDS-PAGE and western blot.

### Wound Closure Assay

Cells were plated in 6-well plates to achieve almost 100% confluence. Cells were scratched with a 100 μL pipette tips, rinsed with PBS, incubated in DMEM supplemented with 10% FBS and 5 μg/mL mitomycin C (Sigma-Aldrich) for 24 h. Cell migration between the scratch areas was monitored at 0 and 24 h, using optical microscope. Migration distance was measured with Image Pro Plus 6.0 (Media Cybemetics, MD, USA).

### Proliferation Assay

Cell proliferation assay was performed using iClick Edu Andy Fluor 647 Flow Cytometry Assay Kit (GeneCopoeia; CA, USA) according to the instruction manual. Briefly, control and MGAT3 over-expressed MDA-MB-231 cells were treated with 40 μM Edu for 6 h. Cells were fixed at appropriated fixation buffer for 15 min at room temperature. After staining and washing with PBS, cells were detected with flow cytometry.

For CCK8 assay, cells were plated in 96-well plate, and incubated with CCK8 (Beyotime Institute of Biotechnology) solution for 4 h. The absorbance in each well was quantified at 450 nm using a microplate reader.

### Clonal Formation Assay

Colony formation was performed as described previously (31). Cells were plated in a 6-cm dish, and cultured for 1–2 weeks until small colonies were clearly observed. Cells were rinsed with PBS, fixed with 4% paraformaldehyde, stained with crystal violet solution, and photographed. Acetic acid solution (10%) was added to dissolve the crystal violet, and absorption at 595 nm was measured.

### Invasion Assay

Invasion assay was performed using cell culture inserts (pore size 8 μm; Corning; New York, USA) as per manufacturer's instructions. 2 × 10^4^ cells in serum-free medium were starved for 24 h, inoculated in upper chamber coated with Matrigel (Corning), and complete medium was added to bottom chamber. After 24 h culture, cells migrated across the membrane were stained with 0.1% crystal violet, and photographed under microscope (magnification 100 ×).

### Flow Cytometry

Cells coated on 24-well plates were detached with trypsin, rinsed with 1 × PBS, blocked with 5% BSA, incubated with FITC-conjugated PHA-E (FL-1121) and SNA (FL-1301) at 4°C for 2 h. Signals from cells were detected by flow cytometry (ACEA NovoCyte; Hangzhou, China), with data acquisition and analysis by the NovoExpress^TM^ software (ACEA bioscience).

### Overexpression of MGAT3

MGAT3 overexpression was performed as previously described (32). Briefly, MGAT3 was cloned into pLVX-AcGFP1-N1 (Takara; Shiga, Japan) lentiviral vector. Lentivirus was packaged in HEK293T cells and collected from medium supernatant. MDA-MB-231 cells were established by infecting lentivirus into cells, and pooled stable transfected cells were selected by adding puromycin. The experiment was performed in duplicate, and the two stable transfected cells were termed as MGAT3-1 and MGAT3-2. Unless otherwise indicated, MGAT3-1 cell line was used for functional study of MGAT3.

### Knockdown of MGAT3

Lentiviral shRNA vector is constructed using pLVX-shRNA2-puro (Takara), and packed into HEK293T cells together with pMD2.G and psPAX2. Virus particles were collected, and used to infect MDA-MB-231 cells. Transfected MDA-MB-231 cells were selected by puromycin. Target sequences are listed below. Target1: 5′-TGTATGGGCTGGACGGCAT-3′; Target2: 5′-CCCAACTTCAGACAGTATGA-3′.

### Lung Colonization Studies

Six- to eight-week-old female Balb/c nu/nu mice were injected with 2 × 10^6^ cancer cells which labeled with luciferase via tail vein. Bioluminescence was determined 6–8 week after injection. Mice were euthanized 8 week after injection, and lungs were fixed, sectioned, and stained with hematoxylin and eosin (H&E) for quantification of metastatic tumor burden.

### Statistical Analysis

All experiments were reproduced at least three times. All data are represented as mean ± standard deviation (s.d.). Two-tailed Student's *t*-test was used for comparison of data sets between two groups, and differences with *p* < 0.05 were considered statistically significant. Statistical analyses were performed using GraphPad Prism V. 7.0 software program. Notations in figures: ^*^p < 0.05; ^**^p < 0.01; ^***^p < 0.001.

## Result

### N-glycan Profiles of Normal and BCa Cells

**Figure 1:**
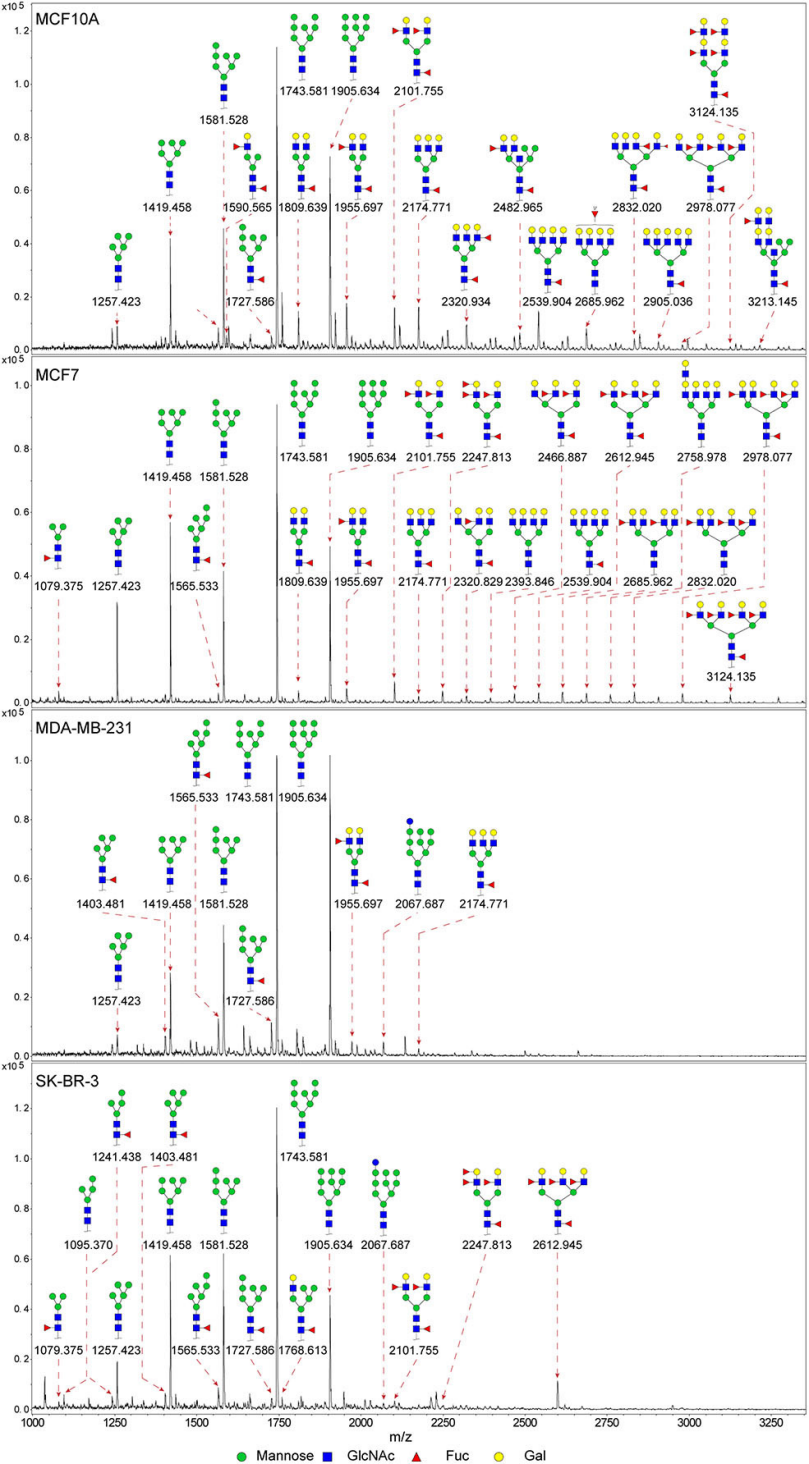
MALDI-TOF-MS spectra of N-glycans. MCF10A, MCF7, MDA-MB-231, and SK-BR-3 cells were cultured in 10 cm dishes, and N-glycans from these cells were isolated as described as M&M. The lyophilized N-glycans were dissolved in methanol/water (1:1, v/v) solution, and an aliquot of the mixture with DHB solution was spotted on an MTP AnchorChip sample target and air-dried. MALDI-TOF-MS was performed in positive-ion mode. Experiments were performed in biological triplicate, and representative N-glycan spectra were shown. Peaks (signal-to-noise ratio >6) were selected for relative proportion analysis. Detailed structures were analyzed using GlycoWorkbench software. Proposed structures were indicated by m/z value.

In previous study, we found the down-regulated expression of bisecting GlcNAc N-glycans in EMT process (24). However, it is not unequivocal if the suppressed bisecting GlcNAc levels is common in BCa cells. We profiled the N-glycans in human mammary epithelial cell line (MCF10A) and human BCa cell lines (MCF7, MDA-MB-231, and SK-BR-3) by MALDI-TOF/TOF-MS analysis. Representative MS spectra of N-glycans were annotated with GlycoWorkbench software (Figure 1). A total of 56 distinct N-glycan structures were identified in the four breast cell lines. MCF10A, MCF7, SK-BR-3, and MDA-MB-231 cells showed 35, 36, 21, and 17 distinct m/z N-glycans, respectively. Nine N-glycan structures were presented in both normal and BCa cells but with different intensities. Six of N-glycan structures, only detected in MCF10A, were annotated as bisecting GlcNAc (Supplementary Table 1).

Relative proportions of different types of N-glycans were calculated and shown in Table 1. We observed that relative proportion of high mannose type of N-glycans were elevated, and which of multi-antennary, and fucosylation were suppressed in three BCa cells comparing to MCF10A cells. Relative proportion of total bisecting GlcNAc in BCa cells were significantly decreased in BCa cells, consist with our previous observation in TGFβ1 induced NMuMG cells.

**Table 1:** Relative proportion of different types of N-glycans in normal and BCa cells.

**Cell type**	**MCF10A**	**MCF7**	**SK-BR-3**	**MDA-MB-231**
**Glycan type**				
High mannose	66.4 ± 3.2%	79.3 ± 6.3%	97.4 ± 1.1%	89.7 ± 1.8%
Complex and hybrid	33.6 ± 1.7%	20.7 ± 6.6%	2.6 ± 0.7%	10.3 ± 1.5%
Bi-antennary	13.9 ± 1.6%	8.1 ± 2.0%	1.1 ± 1%	4.4 ± 1.3%
Multi-antennary	12.2 ± 0.6%	16.4 ± 7.1%	0.1 ± 0.1%	2.2 ± 0.3%
Bisecting GlcNAc	18.9 ± 3.1%	6.4 ± 2.0%	0.7 ± 0.6%	6.7 ± 1.1%
Fucosylation	35.0 ± 3.1%	20.8 ± 6.0%	9.5 ± 3.2%	18.5 ± 0.8%

### Comparison of Glycopattern in Normal and BCa Cells by Lectin Microarray

**Figure 2:**
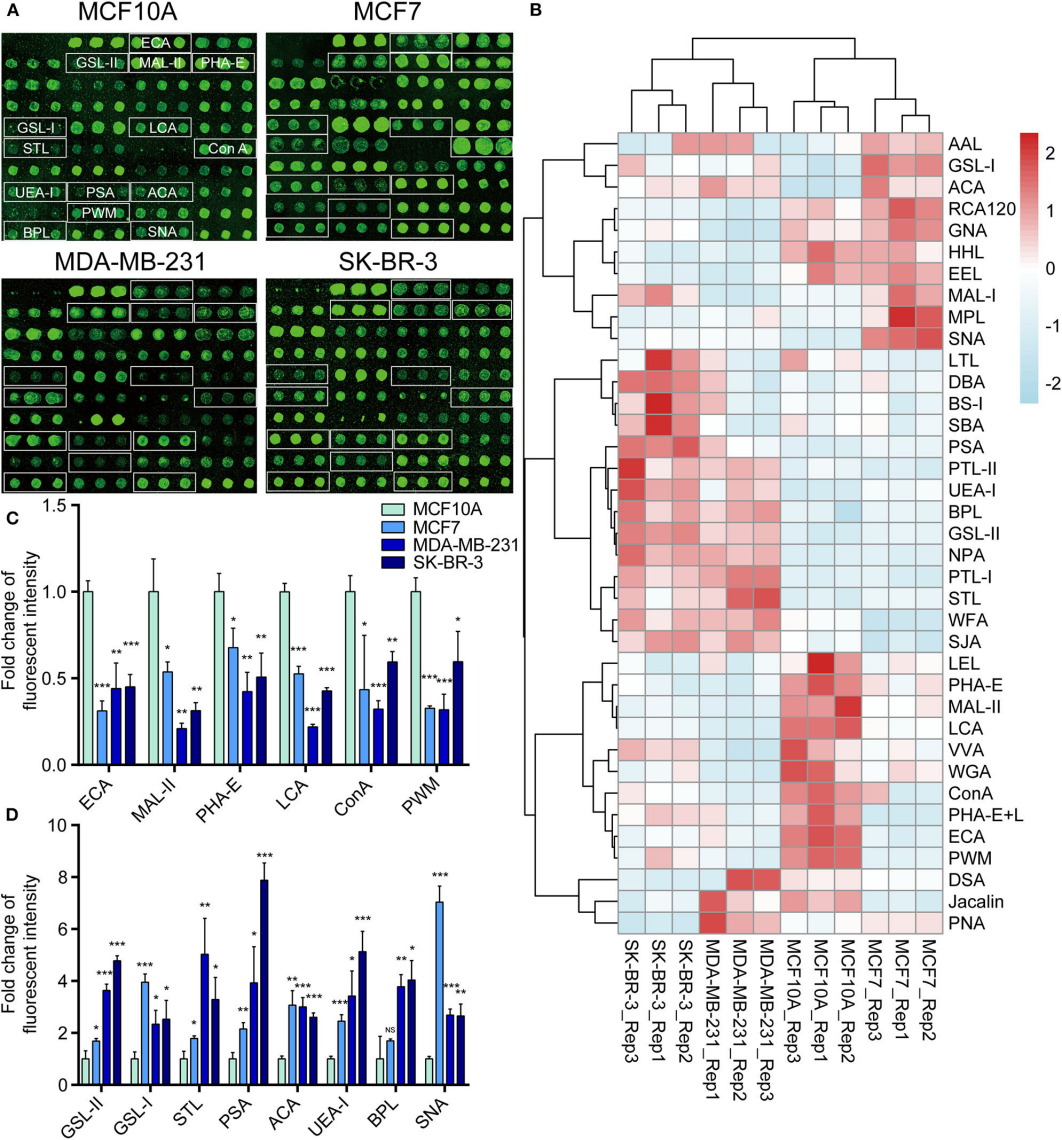
Variation of glycan pattern recognized by lectin microarrays. **(A)** Fluorescence intensities of 37 lectins from the MCF10A, MCF7, MDA-MB-231, and SK-BR-3 cells were scanned. **(B)** Variation of expression of glycans recognized by 37 lectins as a heatmap. Red: fluorescence signal activation. Blue: signal inhibition. **(C)** Relative fluorescence intensities of downregulated glycopatterns recognized by 6 lectins (using cut-offs of *p* < 0.05, fold change *p* < 0.67) in BCa cells comparing to normal breast cells. **(D)** Relative fluorescence intensities of up-regulated glycopatterns recognized by 7 lectins (using cut-offs of *p* < 0.05, fold change *p* >1.5). *p < 0.05; **p < 0.01; ***p < 0.001; NS, *p* > 0.05.

To further investigate the differentially expressed glycopatterns in above four cell lines, lectin microarray analysis was performed. The significant changes in the glycopatterns recognized by lectin were labeled by white boxes in Figure 2A, and corresponding glycan structures were shown in Table 2. Complete hierarchical clustering and visualization were performed with the heatmap package in R software, and clustered side-by-side in the dendrogram (Figure 2B). Significant changes (>1.5-fold or <0.67-fold; *p* < 0.05) of glycopatterns recognized by 14 different lectins were presented (Figures 2C,D). Among them, six glycopatterns including LacNAc structure recognized by lectin ECA, Sia α2-3Gal recognized by lectin MAL-II, bisecting GlcNAc recognized by PHA-E, Fucα1-6GlcNAc (core fucosylated) recognized by LCA, branched and terminal Man or terminal GlcNAc recognized by Con A, and GlcNAc recognized by PWM were suppressed, in BCa cells compared to MCF10A cells. Eight glycan structures including terminal GalNAc and Gal recognized by GSL-I, GlcNAc, and galactosylated N-glycans recognized by GSL-II, (GlcNAc)n recognized by STL, Fucα-N-acetylchitobiose-Man recognize by PSA, T antigen recognized by ACA, H antigen recognized by UEA-I, Galβ1-3GalNAc recognized by BPL, and Sia2-6Gal recognized by SNA were elevated in BCa cells.

**Table 2:** Glycopatterns changes between normal and BCa cells as determined by lectin microarray analysis.

**Lectin**	**Binding structure**	**MCF7/MCF10A**	**MDA-MB-231/MCF10A**	**SK-BR-3/MCF10A**
		**Ratio**		
AAL	Terminal Fucα-1,6GlcNAc 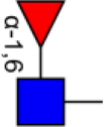 Fucα-1,3Galβ-1,4GlcNAc 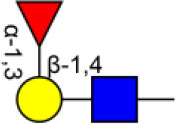	2.76^NS^	2.15^*^	1.27^NS^
ACA	Galβ1-3GalNAcα-Ser/Thr 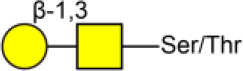	3.07^***^	3.00^**^	2.60^***^
BPL	Galβ1-3GalNAc 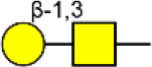	1.69^**^	3.78^NS^	4.03^*^
BS-I	α-Gal, α-GalNAc 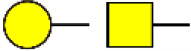	0.90^NS^	1.00^NS^	1.58^NS^
ConA	Branched and terminal Man 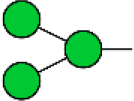 Terminal GlcNAc 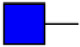	0.43^***^	0.32^*^	0.59^**^
DBA	GalNAcα-Ser/Thr 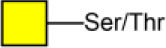 GalNAcα1-3Gal 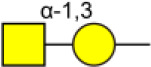	1.05^NS^	1.02^NS^	1.40^***^
DSA	GlcNAc 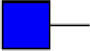	0.73^NS^	1.45^**^	0.08^***^
ECA	Galβ-1,4GlcNAc 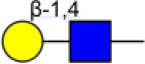	0.31^**^	0.44^***^	0.45^***^
EEL	Galα1-3(Fucα1-2)Gal 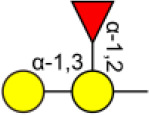	1.07^*^	0.52^NS^	0.61^*^
GNA	Terminal α-1,3 Man 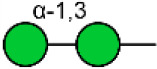	1.27^***^	0.38^NS^	0.88^NS^
GSL-I	αGalNAc, αGal 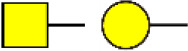 GalNAcα-Ser/Thr (Tn) 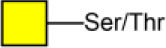	3.96^*^	2.34^***^	2.53^*^
GSL-II	GlcNAc 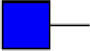 Galactosylated N-glycans 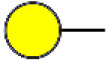	1.69^***^	3.64^*^	4.78^***^
HHL	Non-substituted α-1,6 Man 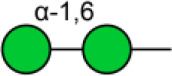	0.89^**^	0.48^NS^	0.52^**^
Jacalin	Galβ1-3GalNAcα-Ser/Thr (T) 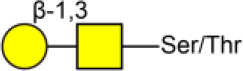 GalNAcα-Ser/Thr (Tn) 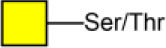	0.55^NS^	0.97^***^	0.61^**^
LCA	Fucα-1,6GlcNAc (core) 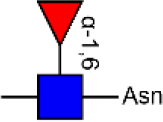	0.53^***^	0.22^***^	0.43^***^
LEL	Poly-LacNAc 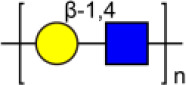 (GlcNAc)n 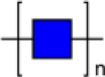	0.79^NS^	0.73^NS^	0.66^NS^
LTL	sLe^x^, Lex 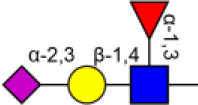 , 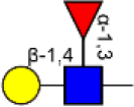 Fucα-1,3GlcNAc (core) 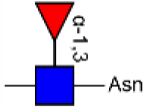	0.57^NS^	0.68^*^	1.23^NS^
MAL-I	Galβ-1,4GlcNAc 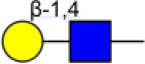	1.51^***^	0.68^*^	1.43^*^
MAL-II	Siaα2-3Galβ1-4Glc(NAc) 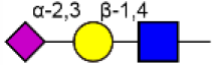	0.54^**^	0.21^*^	0.31^**^
MPL	αGalNAc 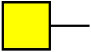	2.75^NS^	1.28^*^	0.83^NS^
NPA	Non-substituted α-1,6Man 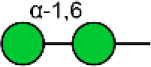	1.09^***^	2.96^NS^	3.31^**^
PHA-E	Bisecting GlcNAc and bi-antennary N-glycans 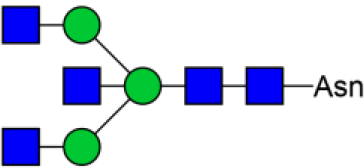	0.68^**^	0.42^*^	0.51^**^
PHA-E+L	Bisecting GlcNAc and bi-antennary N-glycans 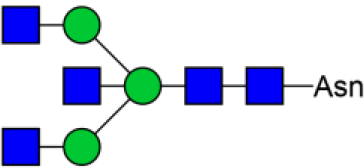 Tetra-antennary complex-type N-glycan 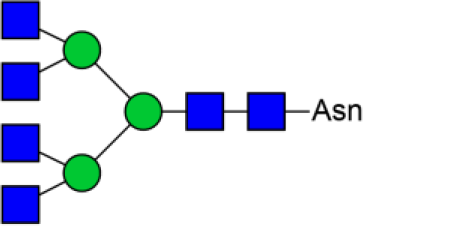	0.46^*^	0.66^***^	0.81^*^
PNA	Galβ1-3GalNAcα-Ser/Thr (T) 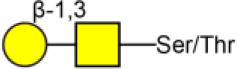	1.14^*^	1.43^*^	0.63^**^
PSA	Fucα-N-acetylchitobiose-Man 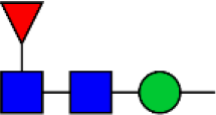	2.15^*^	3.93^**^	7.88^***^
PTL-I	αGalNAc and Gal 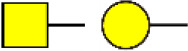	0.95^***^	2.18^NS^	1.85^**^
PTL-II	Gal 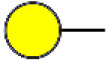	0.89^**^	1.32^NS^	1.42^*^
PWM	GlcNAc 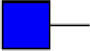	0.33^***^	0.32^***^	0.60^*^
RCA120	Gal, GalNAc 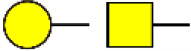	1.42^**^	0.29^*^	0.57^**^
SBA	Terminal GalNAc (especially GalNAcα1-3Gal) 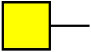	0.91^NS^	0.82^NS^	1.42^*^
SJA	Terminal GalNAc and Gal 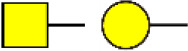	0.11^*^	2.01^*^	2.12^*^
SNA	Sia2-6Galβ1-4GlcNAc 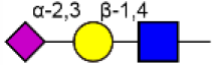	7.04^***^	2.69^***^	2.65^**^
STL	(GlcNAc)n 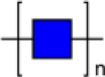	1.78^**^	5.03^*^	3.29^*^
UEA-I	Fucα1-2Galβ1-4Glc(NAc) 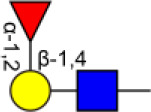	2.45^*^	3.42^***^	5.13^***^
VVA	GalNAc 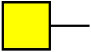 GalNAcα-Ser/Thr (Tn) 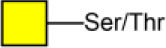	0.82^*^	0.64^NS^	0.94^NS^
WFA	GalNAcα/β1-3/6Gal 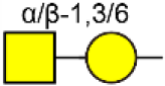	0.28^*^	1.48^***^	1.40^*^
WGA	Multivalent Sia 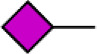 (GlcNAc)n 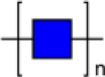	0.70^**^	0.36^NS^	0.60^*^

*NS, not significant*;

* *p < 0.05*;

** p < 0.01; and

*** *p < 0.001*.

**Figure 3:**
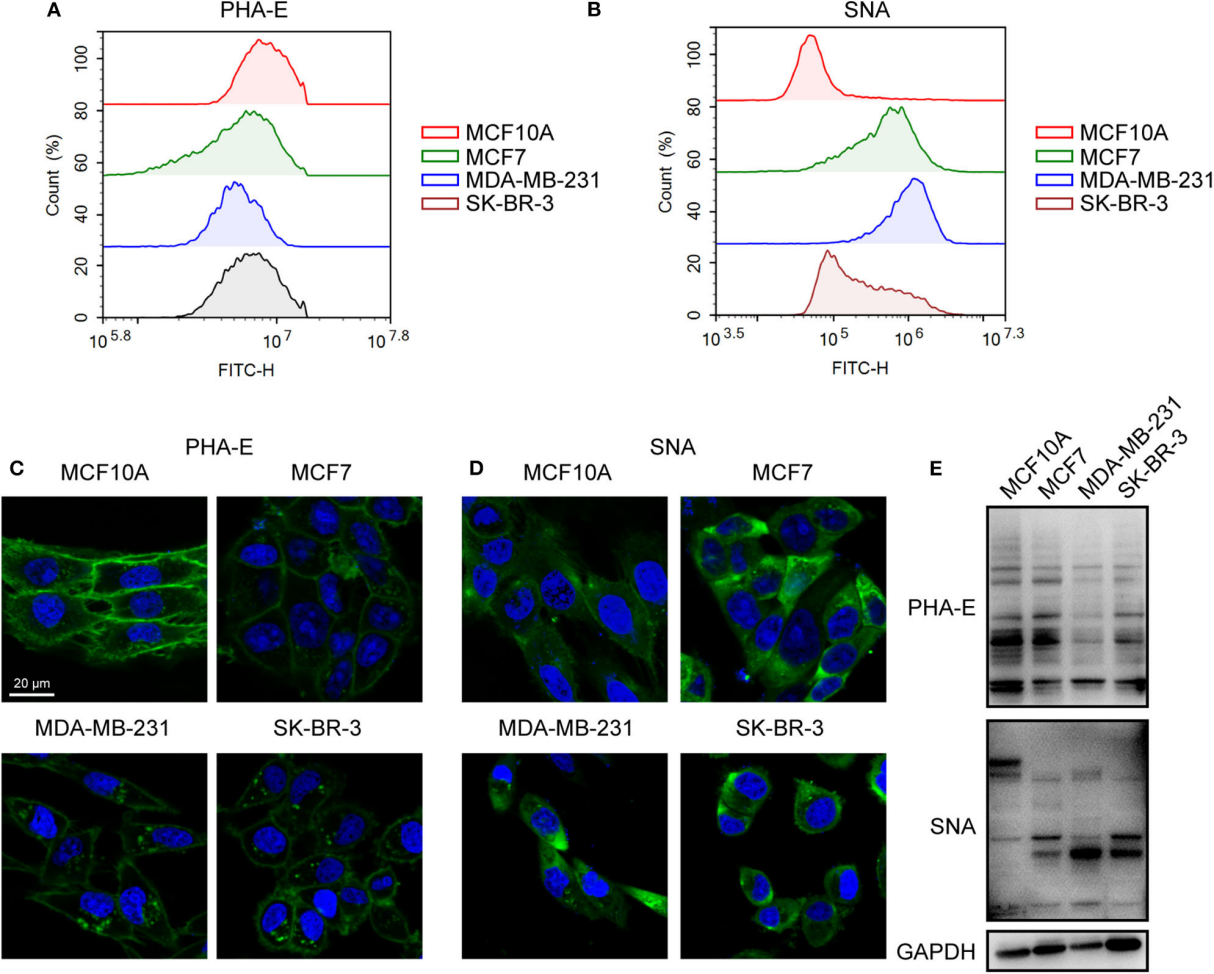
Altered levels of glycan pattern in BC cells. Levels of bisecting GlcNAc **(A)** and α2-6 linked sialic acids **(B)** in normal and BC cells by flow cytometry. Levels of bisecting GlcNAc **(C)** and α2-6 linked sialic acids **(D)** by lectin staining. **(E)** Levels of bisecting GlcNAc and α2-6 linked sialic acids by lectin blot.

To validate the lectin microarray results, flow cytometry analysis of binding lectin showed that bisecting GlcNAc levels (recognized by PHA-E) were suppressed, and α2,6-sialic acid levels (recognized by SNA) were elevated in MCF7, MDA-MB-231 and SK-BR3 comparing to MCF10A, consistent with lectin microarray (Figures 3A,B). Furthermore, results of lectin staining and lectin blot using PHA-E and SNA also indicated the suppressed bisecting GlcNAc levels and elevated α2,6-sialic acid levels in BCa cells (Figures 3C–E).

Together, combined results of mass spectrometry, lectin microarray, flow cytometry analysis, and lectin staining revealed elevated levels of bisecting GlcNAc and suppressed levels of α2,6-sialic acids in BCa cells.

### Identification of Glycoproteins With Bisecting GlcNAc Structures

**Figure 4:**
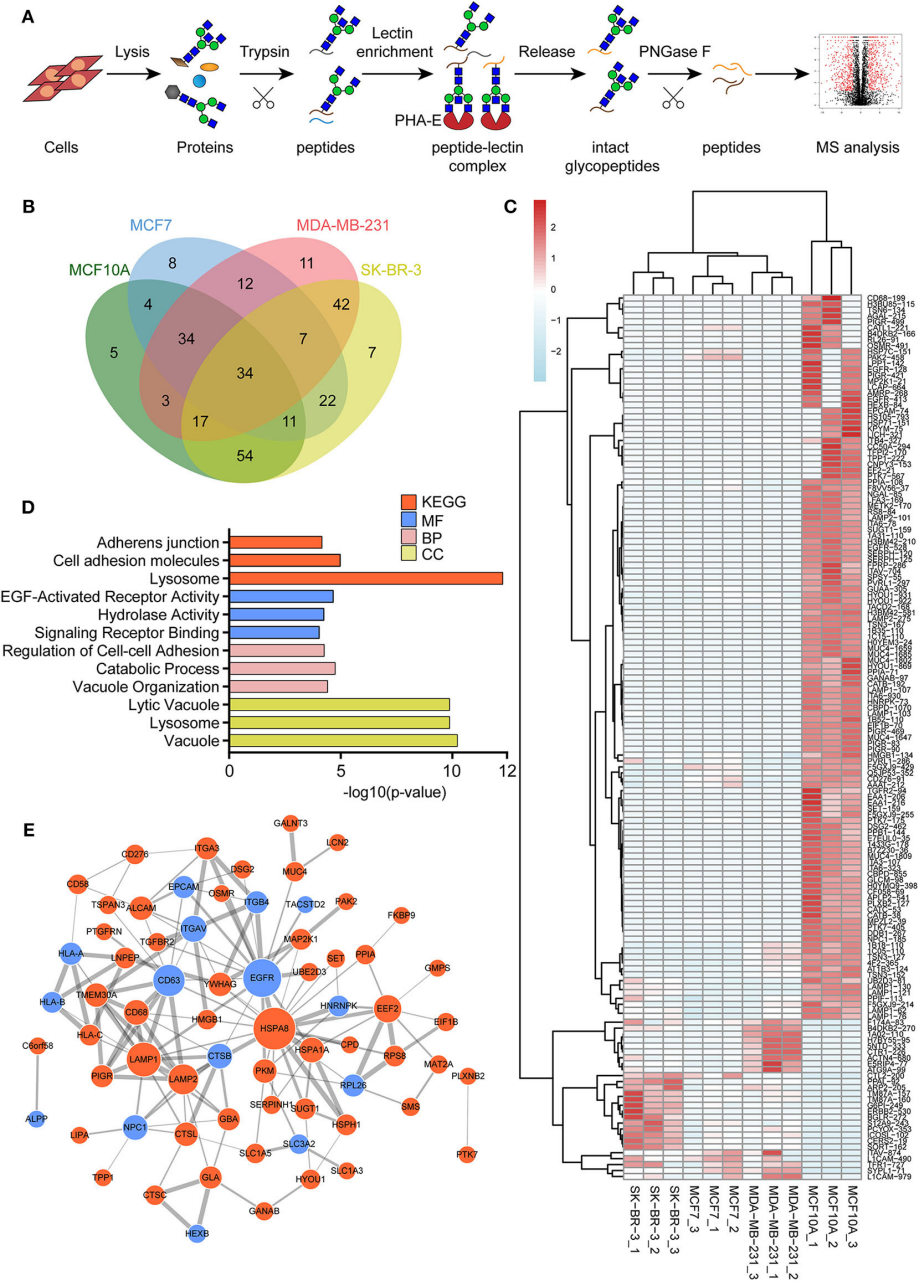
Identification of glycoproteins bearing bisecting GlcNAc. **(A)** Workflow of glycoproteomic analysis by combination of PHA-E enrichment and LC-MS/MS. **(B)** Venn diagram of numbers of identified glycoproteins from MCF10A, MCF7, MDA-MB-231, and SK-BR-3 cells. **(C)** Heatmap of significantly regulated glycopeptides with bisecting GlcNAc. Each colored cell on the map corresponds to a concentration value of the glycopeptide with identified bisecting GlcNAc glycosylation site. **(D)** Functional classification of differentially regulated glycoproteins bearing bisecting GlcNAc using Cytoscape plugin BiNGO based on universal GO and KEGG annotation terms. **(E)** Functional network analysis of differentially regulated glycoproteins using Cytoscape software. Red and blue nodes represented up- and down-regulated glycoproteins. Nodes with more interaction neighbors were displayed in larger size, and edges with larger combined scores were displayed in large size.

To identify the potential glycoproteins bearing bisecting GlcNAc, proteins from above four cell lines were denatured, alkylated, and digested with Lys C and trypsin. Target glycopeptides were enriched with lectin PHA-E-agarose and subjected to mass spectrometry (Figure 4A). A total 504 glycopeptides, which cover 271 glycoproteins, were identified from four cell lines, and shown as the Venn diagram (Figure 4B). These glycopeptides between normal and BCa cells were further comparatively quantified. A number of 150 differentially expressed glycopeptides (fold change>1.5 or <0.67, *p* < 0.05) from 112 glycoproteins were identified, and visualized as a “heatmap” (Figure 4C, Supplementary Table 2). These results indicated that there were characteristic differences in the glycopeptides with bisecting GlcNAc among normal and BCa cells.

Next, gene ontology (GO) terms enrichment analysis of differentially expressed glycoproteins were performed by the Cytoscape plugin BiNGO (Figure 4D). Notably, glycoproteins with bisecting GlcNAc structures were annotated as residing in vacuole, lysosome, and lytic vacuole. Regarding biological processes, identified glycoproteins were mainly involved in catabolic process, regulation of cell-cell adhesion and vacuole organization. With regard to molecular functions, target glycoproteins were enriched in EGF-activated receptor activity, hydrolase activity, and signaling receptor binding. Most significantly enriched KEGG pathway was adhesion junction, cell adhesion molecules and lysosome.

The relationship between the differentially expressed glycoproteins were revealed by the string database (https://string-db.org/). Interactions between them were shown using Cytoscape (Figure 4E). We noticed that HSPA8, EGFR, LAMP1, CD63, and LAMP2 represent main hubs with highest degree scores. As EGFR plays an essential role during BCa progression, EGFR-associated subnetwork was extracted, as shown in Supplementary Figure 1. Down-regulated glycoproteins bearing bisecting GlcNAc, including CTSB, CD63, EPCAM, integrin β4, and TACSTD2 showed clear interactions with EGFR.

### The Confirmation of Bisecting GlcNAc Modification on EGFR

**Figure 5:**
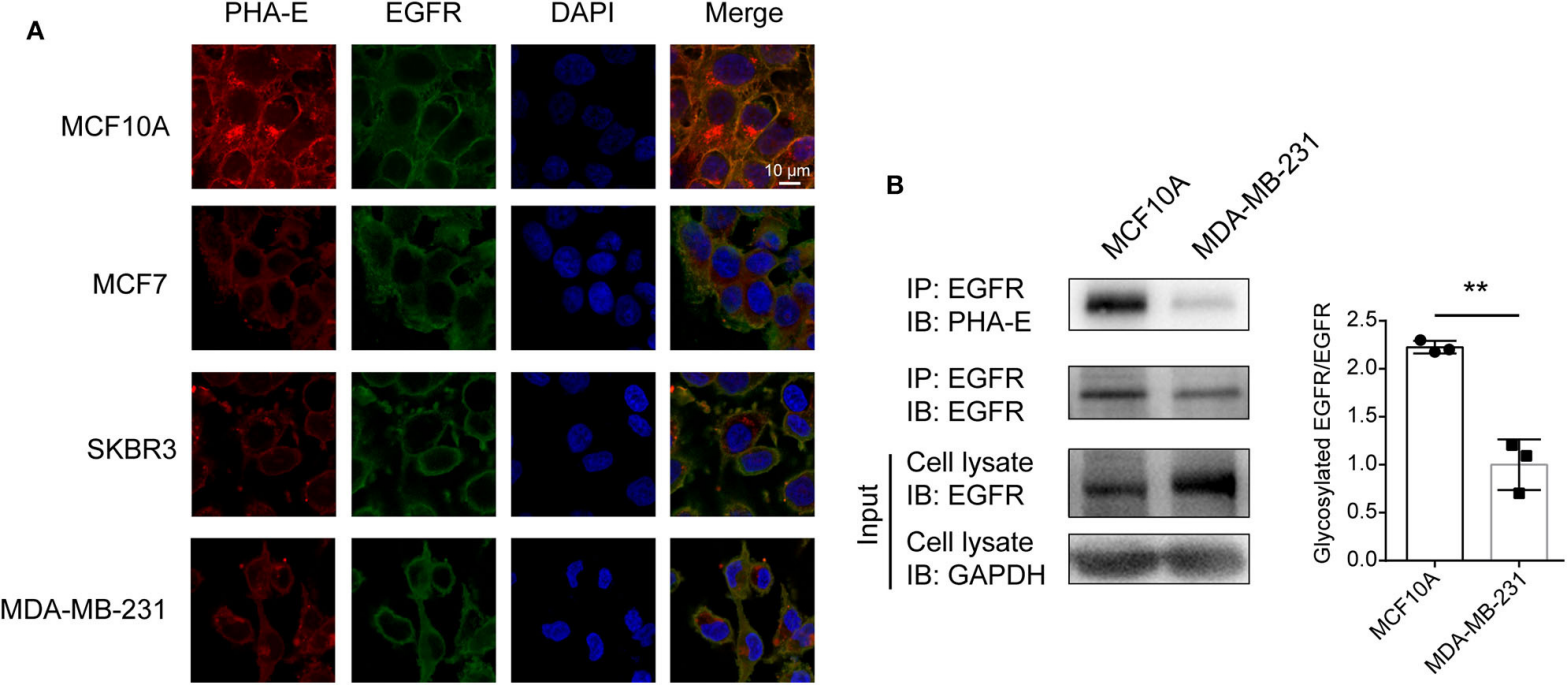
Levels of bisecting GlcNAc on target glycoproteins. **(A)** Altered expression of EGFR and integrin β4 bearing bisecting GlcNAc. Antibody against EGFR and integrin (green) β4, PHA-E lectin (red), and DAPI (blue) were applied, and immunostaining was performed as described as M&M. Fluorescence signals were photographed using confocal microscope. **(B)** Expression of target glycoproteins with bisecting GlcNAc by immunoprecipitation. MCF10A and MDA-MB-231 cell lysate were subjected to immunoprecipitation with antibody against EGFR **(B)**, and immunoprecipitates were subjected to immunoblot analysis. **p < 0.01.

To confirm the differential abundance and cellular location of glycoproteins with bisecting GlcNAc detected in BCa cells by mass spectrometry, immunofluorescence assay was performed. The results showed that EGFR and bisecting GlcNAc were distributed and co-localized on cell membrane (Figure 5A). Lower expression of EGFR was detected in whole cell lysates of MDA-MB-231 compared to which of MCF10A cells (input of Figure 5B). Immunoprecipitation showed significantly decreased levels of bisecting GlcNAc on similar levels of immunoprecipitated EGFR (Figure 5B).

### The Effect of Bisecting GlcNAc on EGFR Function

**Figure 6:**
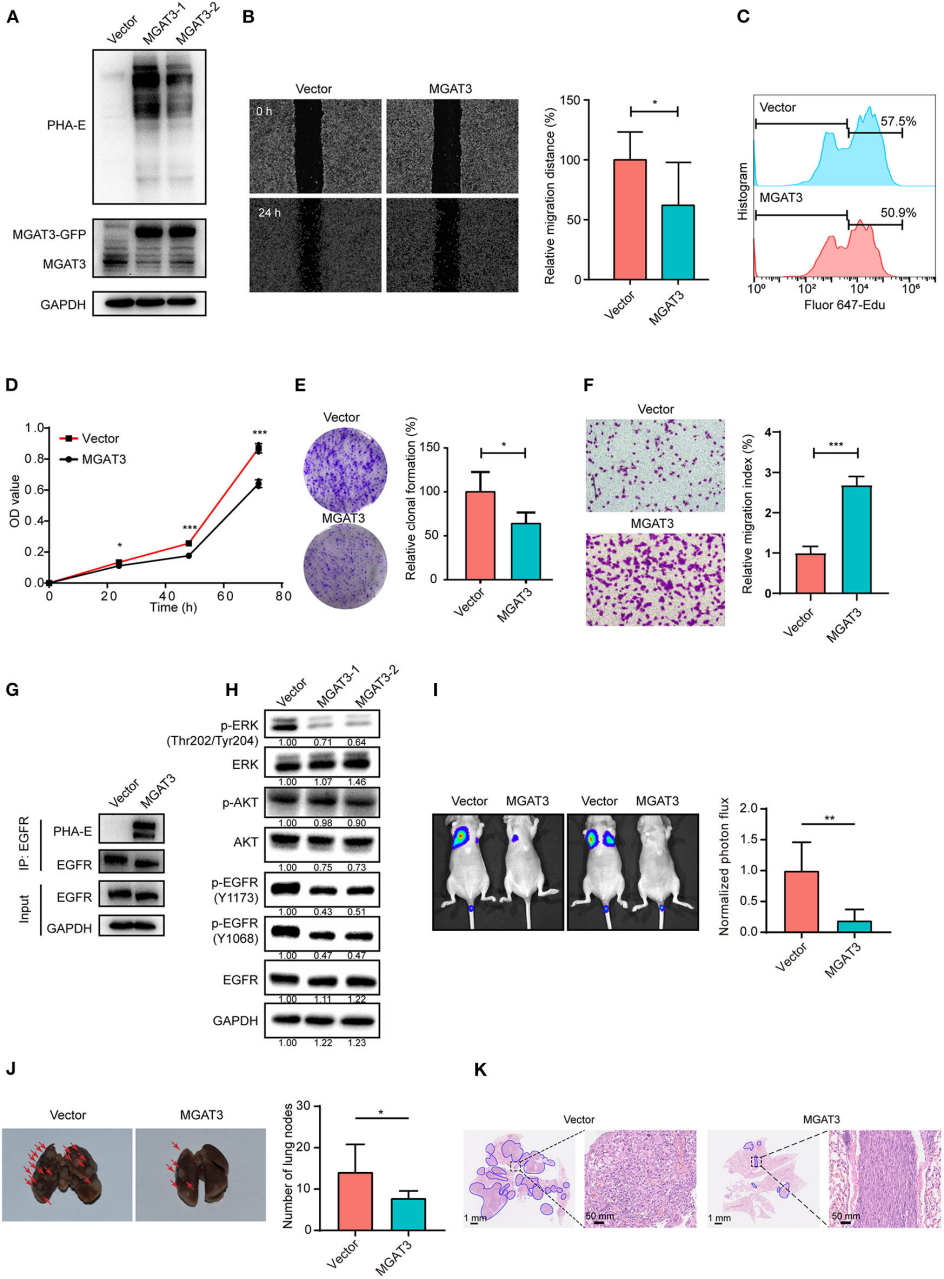
Effects of bisecting GlcNAc on migration, proliferation, clonal formation, and EGFR/ERK signaling activation. **(A)** Expression of MGAT3 and levels of bisecting GlcNAc in control (Vector) and MGAT3-overexpression MDA-MB-231 cells (two transfectants, termed MGAT3-1 and 2). **(B)** Migratory ability of MGAT3 and vector transfectants assessed by wound assay. Transfectant MGAT3-1 was used for following functional assay analysis. Cell monolayers were scratched with pipette tip. Cells were washed with ice-cold PBS and photographed at 0 and 24 h (100 × magnification). *p < 0.05. Cell proliferation of MGAT3 and vector transfectants by EdU incorporation assay **(C)** and CCK8 assay **(D)**. Cells were incubated with 40 μM Edu for 6 h, then fixed with paraformaldehyde, stained and subjected to flow cytometry analysis. ***p < 0.001. **(E)** Colony formation of MGAT3 and vector transfectants. Two thousand five hundred cells were cultured in 6-cm dishes for 1–2 weeks, fixed, stained with crystal violet solution, and photographed. Acetic acid was used to dissolve crystal violet, and OD 595 was determined. *p < 0.05. **(F)** Invasion assay of MGAT3 and vector transfectants. 2 × 10^4^ cells were plated in upper chamber coated with Matrigel. After 24 h culture, cells migrated across the membrane were stained with 0.1% crystal violet, and photographed under microscope (magnification 100×). ***p < 0.001. **(G)** Expression of EGFR with bisecting GlcNAc by immunoprecipitation in MGAT3 and vector transfectants. MGAT3 and vector transfectants lysate were subjected to immunoprecipitation with antibody against EGFR, and immunoprecipitates were subjected to immunoblot analysis. **(H)** Activation of EGFR associated signaling pathway in MGAT3 and vector transfectants by western blot. **(I)** Luciferase activity of nude mice at week 8 following injection of MGAT3-overexpressing MDA-MB-231 and parental cells (each *n* = 6 mice). **p < 0.01. **(J)** Representative photographs and metastatic node numbers of lungs from nude mices injected with MGAT3-overexpressing MDA-MB-231 and parental cells. *p < 0.05. **(K)** Hematoxylin and eosin (H&E) staining of lungs from nude mices injected with MGAT3-overexpressing MDA-MB-231 and parental cells.

To investigate the functional role of bisecting GlcNAc on target glycoproteins in BCa cells. MGAT3 gene was cloned and introduced into MDA-MB-231 cells (two transfectants termed MGAT3-1 and MGAT3-2). The enhanced MGAT3 expression and bisecting GlcNAc levels were validated by western blot (Figure 6A). Scratch wound assay revealed that migratory ability of MDA-MB-231 cells was decreased by introduction of bisecting GlcNAc (Figure 6B). Compared to the vector control, MGAT3 transfectant presented the suppressed proliferation by Edu incorporation assay and CCK8 assay (Figures 6C,D), decreased clonal formation (Figure 6E), and invasion ability (Figure 6F). Immunoprecipitation with antibody against EGFR showed significantly increased levels of bisecting GlcNAc on equal amounts of immunoprecipitated EGFR (Figure 6G), which also demonstrated EGFR as the target glycoprotein of bisecting GlcNAc. Notably, MGAT3 overexpression did not affect the total expression of EGFR, but decreased the levels of EGFR phosphorylation at Y1068 and Y1173 residues (Figure 6H). Furthermore, levels of ERK phosphorylation was decreased at T202/Y204 residues, and AKT phosphorylation was not changed (Figure 6H). MGAT3 was also introduced into another breast cancer cell line BT549 (Supplementary Figure 2A), and overexpression of MGAT3 resulted in decreased proliferation, migratory ability and clonal formation (Supplementary Figures 2B–D). We further silenced MGAT3 expression in MDA-MB-231 cells, shown as Supplementary Figure 2E. Cell proliferation, migratory ability and clonal formation were increased in MGAT3-shRNA transfectant (Supplementary Figures 2F–H). To further evaluate the effect of bisecting GlcNAc on migration and invasion *in vivo*, MGAT3-overexpressing MDA-MB-231 and parental cells were injected into nude mice via the tail vein. As expected, incidence, numbers, and areas of lung metastasis nodules was significantly decreased introduction of bisecting GlcNAc (Figures 6I–K). The results showed that bisecting GlcNAc could retard the cancerous phenotype of BCa cells by suppressing EGFR/ERK signaling.

## Discussion

Glycosylation is implicated in protein folding and stability, cell-cell interaction, angiogenesis, immune modulation, and cell signaling in normal and malignant cells (33). Aberrant glycosylation is an established hallmark of BCa such as CA 16-3, carcinoembryonic antigen (CEA). In our previous study, bisecting GlcNAc levels and MGAT3 expression were decreased in TGFβ-induced EMT of NMuMG cells and hypoxia-induced EMT of MCF7 and MDA-MB-231 cells. In this study, we used an integrated strategy (MALDI-TOF/TOF-MS in combination with lectin microarray) to profile N-glycan alterations in BCa cells comparing to normal cells. Suppressed levels of multi-antennary, bisecting GlcNAc and fucosylation, and elevated levels of high mannose type N-glycans were observed in BCa cells. Theoretically, in the process of N-glycan biosynthesis, bisecting GlcNAc inhibits the catalytic activity of FUT8 and GlcNAcT-V (34). Therefore, reduced levels of bisecting GlcNAc may elevate the levels of multi-antennary structures and fucosylation. The discrepancy may result from preferential action FUT8 and GlcNAcT-V prior to MGAT3. The elevation of high-mannose N-glycans (Man9, m/z 1905.630) were observed in BCa patient serum (35), consistent with increased levels of high-mannose N-glycans in BCa cells in our study, which indicated an incomplete glycosylation process that transform high-mannose N-glycans to complex and hybrid N-glycans. Core fucosylation (catalyzed by FUT8) and β1,6-branching GlcNAc (catalyzed by GlcNAcT-V) have been reported to be enhanced in various cancers (36–38). Core fucosylation plays a key role in the discovery of cancer biomarkers, and core fucosylated α-fetoprotein (AFP) is a well-known tumor marker for hepatocarcinoma (39). β1,6-branching GlcNAc regulates cell surface residency of target proteins, and facilitates the cellular signaling and malignant phenotypes in cancer cells (40). The presence of bisecting GlcNAc may affect the catalytic activity of other glycosyltransferase, thereby resulting in the inhibition of malignant phenotypes.

Modification of bisecting GlcNAc was reported to result in a modulation in biological function of target glycoproteins. To profile the target glycoproteins with bisecting GlcNAc, lectin PHA-E enrichment coupled with nanoLC-MS/MS was performed, and 271 target glycoproteins were identified in normal and BCa cells. Among the identified glycoproteins, EGFR and integrin were previously documented to be decorated with bisecting GlcNAc (11, 41, 42). In PC2 neuronal cells, MGAT3 overexpression contributed to an increase level of bisecting GlcNAc on EGFR, resulting in a significant decrease in EGF binding, EGFR autophosphorylation, ERK activation (43). The introduction of the bisecting GlcNAc to integrin α5 inhibited cell spreading and migration on fibronectin (41). At the molecular level, bisecting GlcNAc might suppress the addition of polyLacNAc on target proteins including EGFR, laminin-332 and integrin. In this case, galectins cannot form the signaling platform, inhibiting both cellular signaling and cell migration (40). In present study, revealed by the protein-protein interaction (PPI) network analysis, EGFR was defined as one of the hub genes with highest degree of connectivity, and interacted with several glycoproteins such as integrin β4 and α3. In confirmation assay, differential expression of EGFR was detected in whole cell lysates of MDA-MB-231 and MCF10A (input of Figure 5B), which was not in line with the similar levels of EGFR evaluated by immunofluorescence (Figure 5A). The discrepancy might result from the differences of the two techniques in quantification. Increased level of bisecting GlcNAc on EGFR lowered the molecular weight of EGFR (Figure 6G), which might result from the changes in glycosylation. Increased level of bisecting GlcNAc also suppressed the migratory ability, cell proliferation and clonal formation ability by suppressing the EGFR/Erk signaling pathway, in MGAT3-overexpressed MDA-MB-231 cells. This data confirmed the inhibitory function of bisecting GlcNAc on EGFR, consistently with other studies (11, 44, 45).

Moreover, in this study, other identified glycoproteins, including lamp1, TGFβRII, HSPA8, etc., were not reported to be decorated with bisecting GlcNAc to date. Lamp1 was a major carrier of polyLacNAc substituted β1,6 branched GlcNAc in melanoma cells. Translocation of lamp1 with polyLacNAc to the surface was closely associated with the metastatic potential (46). PolyLacNAc decoration on lamp1 provides abundant ligands (polyLacNAc) for galectin-3 distributed on the surface of lung vascular endothelium, facilitating lung homing (46, 47). We proposed that bisecting GlcNAc modification on lamp1 might decrease levels of polyLacNAc, and suppress interaction of lamp1 and galectin-3, and result in low metastatic potential. TGFβRI and TGFβRII were reported to be N-glycosylated. Expression of N-acetylglucosaminyltransferase V (MGAT5) enhanced the interaction of galectin-3 and TGFβRII, and delayed the removal of TGFβR by constitutive endocytosis, sensitized mouse cells to multiple cytokines (48). Similarly, bisecting GlcNAc modification might resist the activation to multiple cytokines by weakening the interaction of galectin-3 and TGFβR. Furthermore, recent study showed that bisecting GlcNAc on intact glycopeptides could be recognized by two characteristic ions at low energy HCD spectra (49). This advanced analytical strategy may help to identify more intact glycopeptides with bisecting GlcNAc in future study.

In conclusion, we found decreased levels of bisecting GlcNAc in BCa cells compared to normal breast cells by mass spectrometer and lectin microarray. Using PHA-E-based enrichment coupled with nanoLC-MS/MS, glycoproteins bearing bisecting GlcNAc were identified in various BCa cells. Among identified glycoproteins, levels of bisecting GlcNAc on EGFR were significantly decreased in BCa cells. Introduction of bisecting GlcNAc on EGFR in BCa MDA-MB-231 cells resulted in the reduction of migratory ability, cell proliferation, clonal formation, and suppression of EGFR/Erk signaling. Our study might provide potentially valuable information for BCa diagnosis and treatment.

## Data Availability Statement

The raw data supporting the conclusions of this article will be made available by the authors, without undue reservation, to any qualified researcher.

## Ethics Statement

The study was carried out in accordance with the recommendations of the Institutional Animal Care and Use Committee of Northwest University. The protocol was approved by the Institutional Animal Care and Use Committee of Northwest University.

## Author Contributions

FG and ZT conceived and initiated this project. All experiments described in this paper were performed by LCh, LCa, YW, WX, and JL, who then generated the figures and tables for the paper. LCh, LCa, and ZT wrote the first draft of the manuscript, which was then further edited by FG.

## Conflict of Interest

The authors declare that the research was conducted in the absence of any commercial or financial relationships that could be construed as a potential conflict of interest.

## Supplementary Material

Supplementary Figure 1 Functional network analysis of EGFR-associated subnetwork.

Supplementary Figure 2 Effects of bisecting GlcNAc on migration, proliferation, and clonal formation.

Supplementary Table 1 Proposed structures and their molecular ions in MALDI Spectra of N-glycans from normal and BCa cells.

Supplementary Table 2 Glycopeptides identified bearing bisecting GlcNAc by nanoLC-MS.
