# Supplementary material for: Bisecting N-Acetylglucosamine on EGFR Inhibits Malignant Phenotype of Breast Cancer via Down-Regulation of EGFR/Erk Signaling
Source: Front Oncol. 2020 Jun 16;10:929. doi: 10.3389/fonc.2020.00929 (PMC7308504; doi:10.3389/fonc.2020.00929)
Supplement: Supplementary Figure 1 — Functional network analysis of EGFR-associated subnetwork. [file Data_Sheet_1.docx]

Supplementary Material


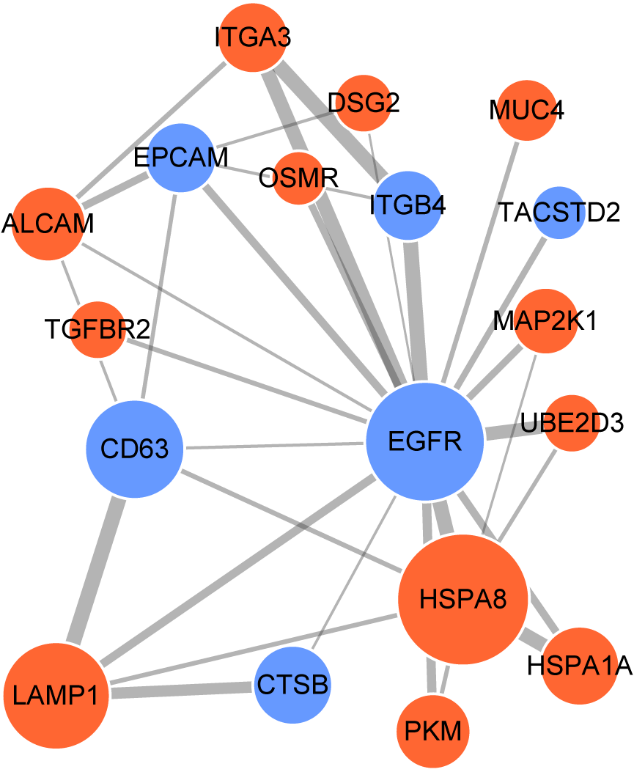


**Supplementary Figure 1.** Functional network analysis of EGFR-associated subnetwork.


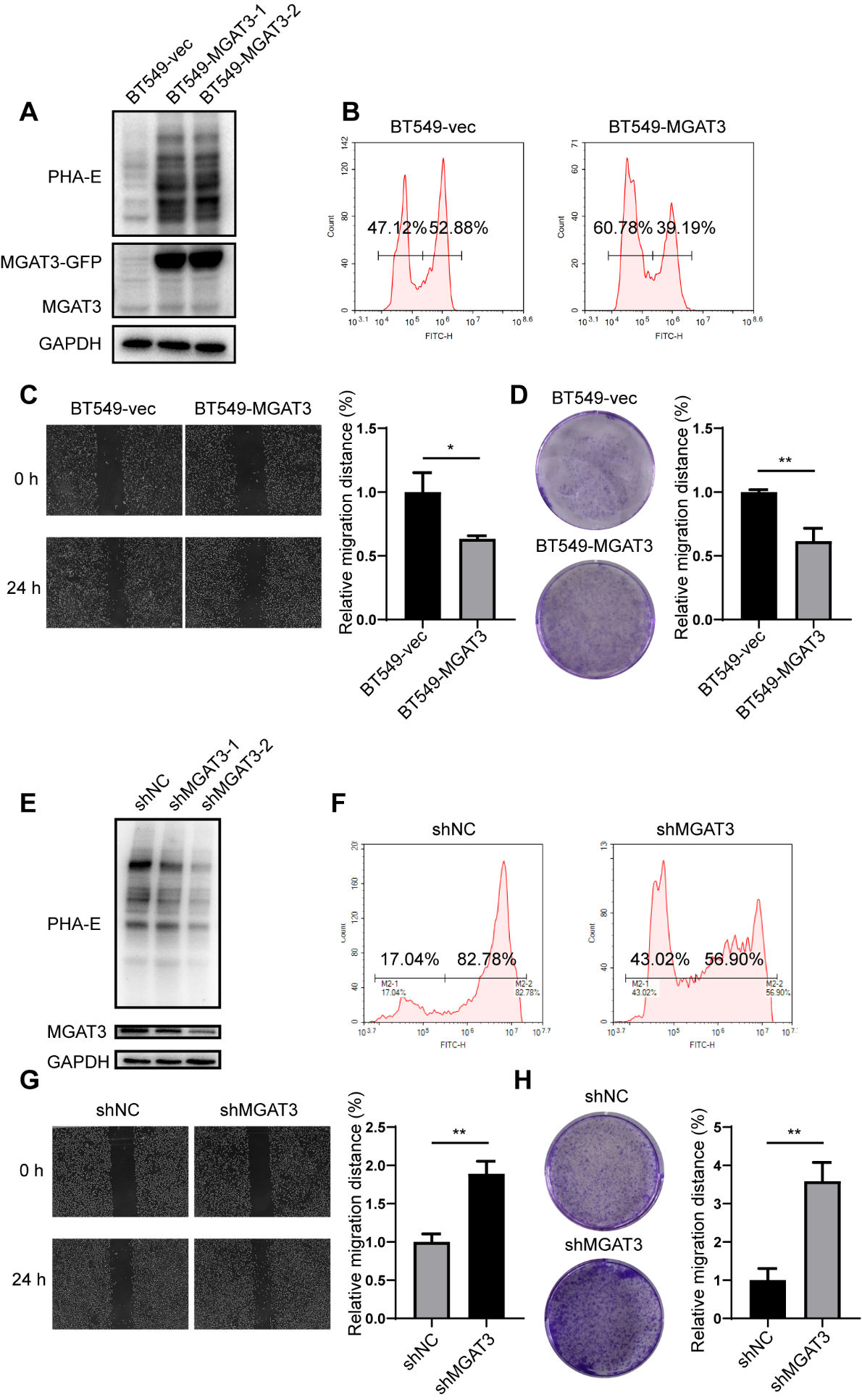


**Supplementary Figure 2.** Effects of bisecting GlcNAc on migration, proliferation, clonal formation. (A) Expression of MGAT3 and levels of bisecting GlcNAc in control and MGAT3-overexpression BT549 cells (two transfectants, termed BT549-MGAT3-1 and 2). (B) Cell proliferation of MGAT3 and vector transfectants by EdU incorporation assay. Transfectant BT549-MGAT3-1 was used for following functional assay analysis. (C) Migratory ability of MGAT3 and vector transfectants assessed by wound assay. *, p<0.05. (D) Colony formation of MGAT3 and vector transfectants. **, p<0.01. (E) Expression of MGAT3 and levels of bisecting GlcNAc in MGAT3-shNC (termed shNC) and MGAT3-shRNA transfected MDA-MB-231 cells (two transfectants, termed shMGAT3-1 and 2). (F) Cell proliferation of shNC and MGAT3-shRNA transfectants by EdU incorporation assay. Transfectant shMGAT3-2 was used for following functional assay analysis. (G) Migratory ability of shNC and MGAT3-shRNA transfectants assessed by wound assay. **, p<0.01. (H) Colony formation of shNC and MGAT3-shRNA transfectants. **, p<0.01.
